# Lake sediment fecal and biomass burning biomarkers provide direct evidence for prehistoric human-lit fires in New Zealand

**DOI:** 10.1038/s41598-018-30606-3

**Published:** 2018-08-14

**Authors:** E. Argiriadis, D. Battistel, D. B. McWethy, M. Vecchiato, T. Kirchgeorg, N. M. Kehrwald, C. Whitlock, J. M. Wilmshurst, C. Barbante

**Affiliations:** 1 Department of Environmental Sciences, Informatics and Statistics, Ca’ Foscari University of Venice, Via Torino 155, 30170 Venezia Mestre, VE Italy; 2 Institute for the Dynamic of Environmental Processes (IDPA-CNR), Via Torino 155, 30170 Venezia Mestre, VE Italy; 3 Department of Earth Sciences and Montana Institute on Ecosystems, Montana State University, PO Box 173840, Bozeman, MT 59717 USA; 4 U.S. Geological Survey, Geosciences and Environmental Change Science Center, MS 980, Denver, CO 80225 USA; 5 Long-Term Ecology Lab, Landcare Research, PO Box 69040, Lincoln, New Zealand; 6 School of Environment, University of Auckland, Private Bag 92019, Auckland, New Zealand

## Abstract

Deforestation associated with the initial settlement of New Zealand is a dramatic example of how humans can alter landscapes through fire. However, evidence linking early human presence and land-cover change is inferential in most continental sites. We employed a multi-proxy approach to reconstruct anthropogenic land use in New Zealand’s South Island over the last millennium using fecal and plant sterols as indicators of human activity and monosaccharide anhydrides, polycyclic aromatic hydrocarbons, charcoal and pollen as tracers of fire and vegetation change in lake-sediment cores. Our data provide a direct record of local human presence in Lake Kirkpatrick and Lake Diamond watersheds at the time of deforestation and a new and stronger case of human agency linked with forest clearance. The first detection of human presence matches charcoal and biomarker evidence for initial burning at c. AD 1350. Sterols decreased shortly after to values suggesting the sporadic presence of people and then rose to unprecedented levels after the European settlement. Our results confirm that initial human arrival in New Zealand was associated with brief and intense burning activities. Testing our approach in a context of well-established fire history provides a new tool for understanding cause-effect relationships in more complex continental reconstructions.

## Introduction

Humans have altered landscapes around the world for thousands of years through their use of fire^1^. However, the intensity and extent of anthropogenic burning in many regions remains unresolved^2^. Most reconstructions of past fire regimes focus on variations in the concentration of charcoal particles preserved in the sediments of lakes and other natural wetlands. Such records disclose the occurrence of fire and variations in the frequency and timing of biomass burning, but they are unable to discern whether ignitions originate from natural or human causes. Finding methods for disentangling naturally caused fires from those deliberately started by people remains one of the great challenges in paleofire science^1,2^. Times and places where past fire activity and vegetation depart from trends that can be explained by climate alone are often used as indirect evidence for anthropogenic burning. Identifying past human presence in particular watersheds has generally relied on archeological data, but few locations offer a clear archeological signal of local fire use. As a result, linking past changes in vegetation and fire with human activity is largely inferential^3,4^.

One of the most dramatic examples of prehistoric anthropogenic burning occurs in New Zealand, where charcoal and pollen records provide incontrovertible evidence of unprecedented fire activity and native forest decline coinciding with the arrival of people at c. AD 1280^5–8^. Results from modeling studies that examine vegetation-fire feedbacks attribute the rapid environmental change to small-scale positive feedbacks between fire and the post-fire establishment of high flammability shrublands that enable subsequent burning^9,10^. Humans, as a new source of ignition, thus triggered a conversion of forest to a more fire-prone shrubland/grassland. This reconstruction, however, is based on charcoal and pollen data alone, and in the absence of archeological evidence of burning it is not possible to determine if humans were present in a particular watershed at the time of a fire event, or if the timing of their activities coincided with the loss of forest^11,12^. Here we provide direct evidence of brief but intense intervals of local human activity in two New Zealand watersheds at the same time as other proxies show fire and deforestation.

**Figure 1:**
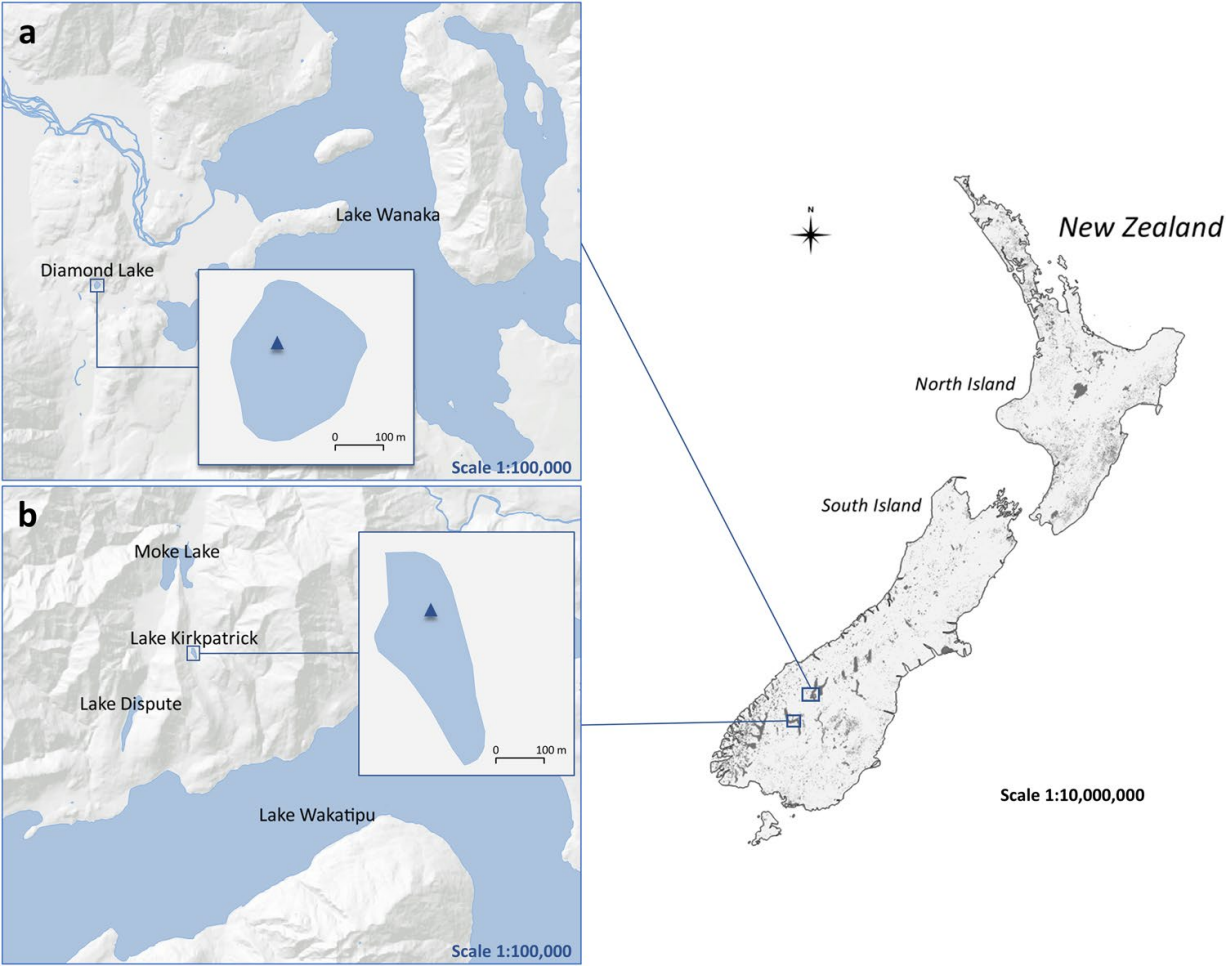
Map of the sampling locations. (**a**) Diamond Lake in the Lake Wanaka area and sampling location. (**b**) Lake Kirkpatrick in the Lake Wakatipu area and sampling location. Contains data sourced from the LINZ Data Service licensed for reuse under CC BY 3.0 (https://creativecommons.org/licenses/by/3.0/nz/).

We utilize specific molecular markers to provide a continuous record of human presence, fire activity and land use in the South Island of New Zealand during the last millennium. We determined polycyclic aromatic hydrocarbons (PAHs), monosaccharide anhydrides (MAs), fecal and plant sterols fluxes in sediment cores from Lake Kirkpatrick (45.03° S, 168.57° E) and Lake Diamond (44.65° S, 168.96° E) (Fig. 1) to compare with pollen and charcoal records and paleoclimate information. The three MA compounds (levoglucosan, mannosan and galactosan) are formed from cellulose and hemicellulose combustion^13^ and the seventeen PAHs (Table S1) are tracers for incomplete combustion of organic matter^14^. MAs and PAHs can travel medium to long distances in association with the fine fraction of aerosol particles^15,16^ and ultimately reach lake sediments through wet and dry deposition^17^. Their signal in lakes complements the macroscopic charcoal (pieces > 125 μm) signal of local fires by providing fire information at regional scales. In Lake Kirkpatrick and Lake Diamond, we also employ two 5β-stanols originating from human feces (coprostanol and epi-coprostanol)^18^ to trace human presence in the catchments. Two Δ^5^-sterols (cholesterol and sitosterol) and two 5α-stanols (cholestanol and sitostanol) are used as additional land-use proxies, as these markers account for terrigenous input to the lake and the chemical conditions of sediments^19,20^ (Table S2).

## Fire Record

**Figure 2:**
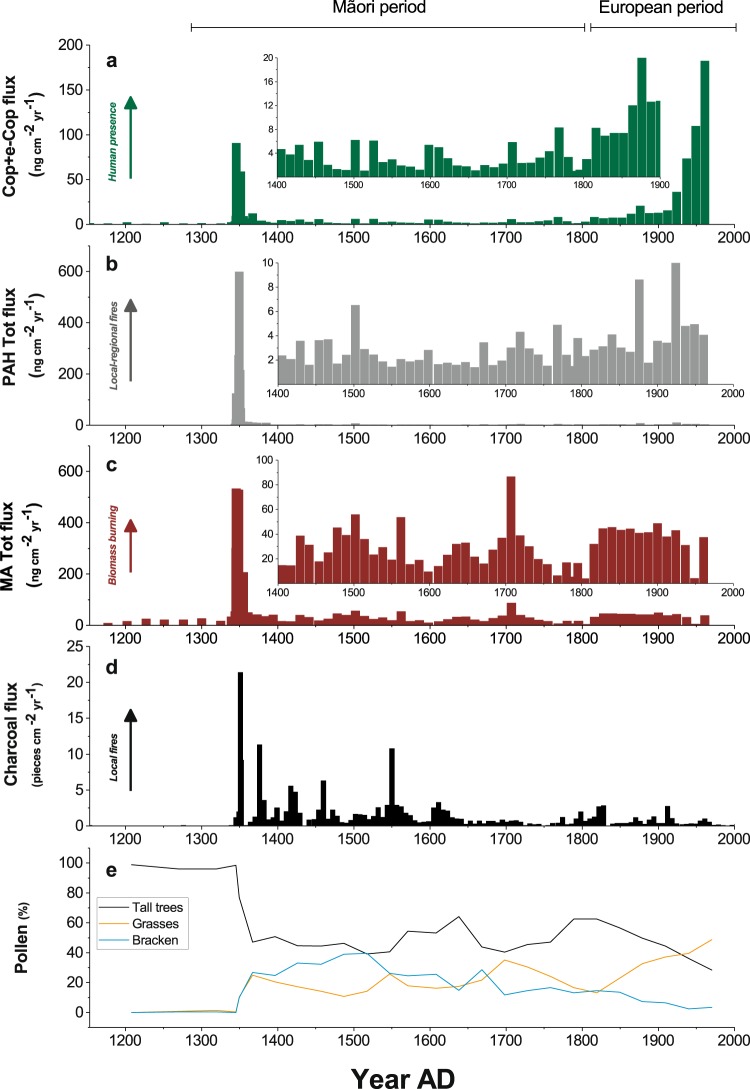
Multi-proxy comparison. (**a**–**c**) Fecal sterols, total PAH and total MA fluxes (ng cm^−2^ yr^−1^) in Lake Kirkpatrick (this study). (**d**,**e**) Charcoal flux (pieces cm^−2^ yr^−1^) and pollen percentages in Lake Kirkpatrick (McWethy *et al*.^6^).

Prior to initial human settlement c. AD 1280, fluxes of charcoal and biomarkers in Lake Kirkpatrick and Lake Diamond were nearly undetectable (Fig. 2). The lack of large charcoal particles (>125 µm in diameter) testifies to the near-absence of fire in the surrounding *Lophozonia*-podocarp forests which is typical of records from the region^5^. The presence of extremely low values of molecular fire tracers (PAH total flux < 1 ng cm^−2^ yr^−1^, MA total flux < 4 ng cm^−2^ yr^−1^) prior to human arrival is attributed to small fires in drier settings in New Zealand or to background atmospheric deposition from distant fires in southeastern Australia and post-depositional processes^21–23^.

The fluxes of all MAs and PAHs abruptly and simultaneously increase in sediments at c. AD 1345–1365 and mark a period of intense or multiple fire events. The peaks occur shortly after Māori arrival and during the Initial Burning Period, which has been identified from charcoal records from Lake Kirkpatrick and other South Island lakes^5,24,25^. Levoglucosan, mannosan and galactosan records clearly indicate that combustion of plant biomass reached a maximum at c. AD 1350 with fluxes of 390, 278 and 66 ng cm^−2^ yr^−1^, respectively. Relative proportions of the three isomers are consistent with emission factors typical of conifer burning^26^.

The PAH pattern (Fig. S1) is consistent with typical PAH profiles obtained from the combustion of biomass including several types of hardwood^27^ and softwood^28–30^. Low molecular weight compounds (Table S1), such as naphthalene, acenaphthylene and acenaphthene (128–154 g mol^−1^), are poorly represented, which is not surprising as they are commonly present in the gaseous phase and relatively more water-soluble and prone to biodegradation than heavier PAHs, which absorb to atmospheric particles^31^. The PAH distribution is dominated by 3- and 4-ring molecules (166–228 g mol^−1^), in particular phenanthrene, fluorene, fluoranthene and pyrene (Fig. S1). These PAH compounds are produced during biomass burning and are involved in the formation of atmospheric particles, eventually incorporated into lake sediments as a result of aerial deposition and surface runoff^17^. At Lake Kirkpatrick, phenanthrene accounted for 43% of total PAHs on average, with concentrations ranging from a few nanograms per gram (dry weight) to a maximum of 212 ng g^−1^, corresponding to a flux of 180 ng cm^−2^ yr^−1^, during the IBP. Heavier compounds (252–278 g mol^−1^) were present only in small to negligible concentrations, and this was not surprising as they are mainly produced by higher temperature processes (e.g. fossil fuel combustion) and associated with coarse particles, less likely to travel far from the source area^17,32,33^.

The abundance and distribution of medium-weight PAHs during the Initial Burning Period is consistent with sustained fires characterized by low oxygen availability and a high flaming to smoldering combustion ratio^34,35^. Based on the scarcity of high molecular weight PAHs, on the thermal stability of detected compounds^36^ and on burning experiments^37,38^, we infer a maximum combustion temperature averaging 400–500 °C. This temperature range was found to maximize the production of 3–4 ring PAHs from biomass combustion^32,39^. Concentrations and trends detected for medium-weight PAHs thus suggest an infrequent low-intensity natural fire regime before the arrival of humans and high-intensity or high-frequency fires during the Initial Burning Period.

The peak in retene, a tracer of combusted coniferous wood and the associated degradation of abietic acid^30^, in the L. Kirkpatrick core (101–112 cm depth) implies an abundance of softwood fuel (Fig. S2). However, the levoglucosan to mannosan ratio suggests an increased hardwood to softwood fuel ratio^40^ at ~AD 1348–1394 (101–112 cm), ~AD 1790–1805 (33–36 cm) and ~AD 1924–1949 (9–13 cm) (Fig. S2). The observed retene record is consistent with the combustion and/or the post-depositional reduction of diterpenoids from softwood species^41^.

After c. AD 1350, the pollen data^5,6^ suggest a shift in the composition of vegetation from native *Lophozonia*-podocarp forest towards more fire-adapted shrubs (e.g., *Leptospermum*), grasses and bracken fern (*Pteridium esculentum*). The biomarker reconstruction agrees with the new fuel types that replaced the podocarp-hardwood forests and the change in fire regime characterized by infrequent or smaller low-intensity fires based on low levels of macroscopic charcoal (Fig. 2)^6^. Such fires would result in lower temperatures and less oxygen depleted conditions, limiting the production of MAs and PAHs, which is strongly dependent on fuel and burning conditions^30,35^, as observed in the post-settlement record.

## Anthropogenic Land Use Record

Unlike the fire tracers, sterols primarily reach lake sediments through runoff^42^, and thus describe inputs from the local watershed. The evidence of human presence within the Lake Kirkpatrick and Lake Diamond catchments is provided by coprostanol - the most abundant sterol in human feces^18^- and epi-coprostanol resulting from the epimerization of coprostanol^19^. In addition, cholesterol and cholestanol are produced by vertebrates, especially mammals including humans^43^, whereas sitosterol (the most abundant sterol in land plants^44,45^) and sitostanol come from the input of terrigenous organic matter to the lake. The degree of sterol to stanol conversion infers redox conditions of the basin^46^ (see supplementary information).

Prior to human settlement, fluxes of fecal sterols in both lakes were near zero, although we measured small background fluxes (at the sub-ng cm^−2^ yr^−1^ level) of coprostanol and epi-coprostanol in all pre-Maori samples. Small amounts of coprostanol were previously detected also in anaerobic soils devoid of a fecal input and related to the presence of microbial species capable of converting cholesterol to 5β-stanols^47,48^, which would provide an explanation for the presence of a natural background in anoxic sediments.

**Figure 3:**
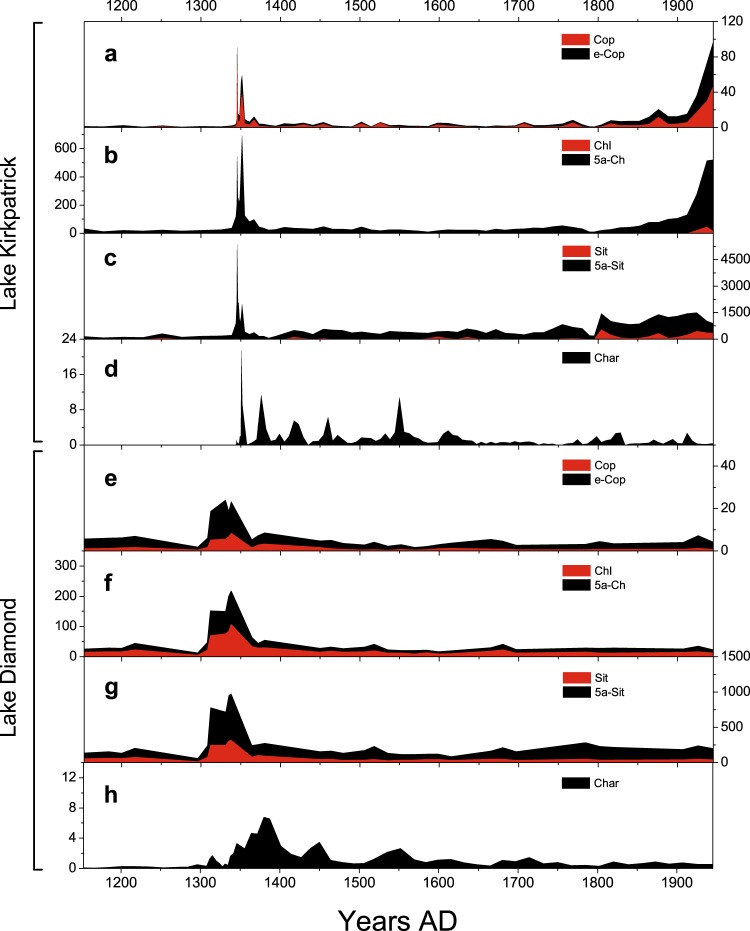
Fire and human presence at Lake Kirkpatrick and Lake Diamond. (**a**–**c**) Sterols in Lake Kirkpatrick (this study). (**d**) charcoal flux in Lake Kirkpatrick (McWethy *et al*.^6^). (**e**–**g**) Sterols in Lake Diamond (this study). (**h**) charcoal flux in L. Diamond (McWethy *et al*.^25^). All sterols fluxes are shown as ng cm^−2^ yr^−1^ and charcoal fluxes as pieces cm^−2^ yr^−1^.

The substantial increase at c. AD 1345–1365 (106–116 cm depth) in Lake Kirkpatrick and at c. AD 1310–1380 (42–55 cm depth) in Lake Diamond (Fig. 3) likely originated from human waste.

A short-lived peak in fecal sterols occurred with the onset of the Initial Burning Period in the Lake Kirkpatrick record, and slightly preceded it at Lake Diamond^25^. The brevity of the peaks at both sites suggests that human activity in the watershed lasted only a few decades. After a large increase in fire activity during the 1300 s, the charcoal influx and fecal sterols values decreased dramatically but remained at about twice the flux of the pre- Māori period. This decline between c. AD 1400 and 1800 likely marks a reduced presence of people in the watershed prior to European colonization, although humans may have been present sporadically or in low numbers.

**Figure 4:**
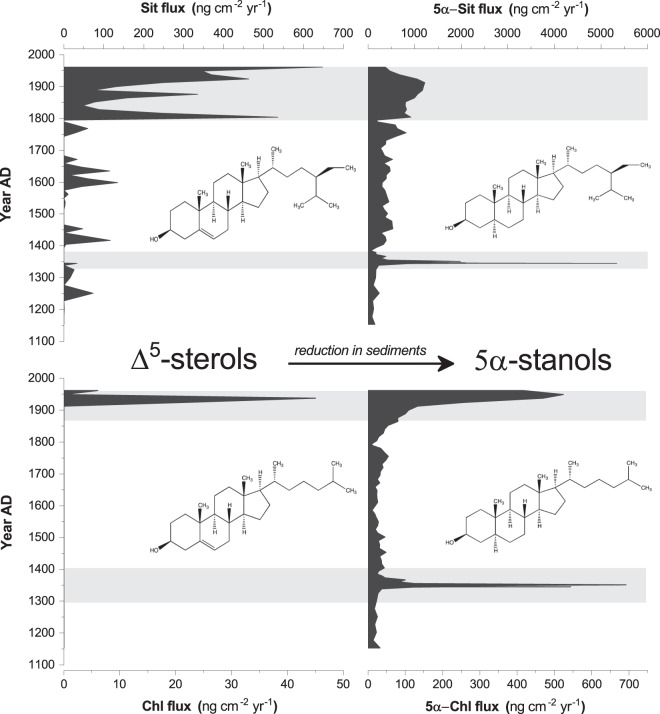
Reduction of Δ^5^-sterols in sediments of Lake Kirkpatrick. (**a**) Sitosterol. (**b**) Sitostanol. (**c**) Cholesterol. (**d**) Cholestanol. All compounds are plotted as fluxes (ng cm^−2^ yr^−1^). The favored reaction pathway converts Δ^5^-sterols (Sit, Chl) to 5α corresponding stanols (5α-Sit, 5α-Chl).

An increase in sedimentation rate at Lake Kirkpatrick at ~AD 1340–1350 (108–118 cm depth) and Lake Diamond at ~AD 1300–1450 (35–56 cm depth) is consistent with increased erosion following forest clearance. In addition, the high levels of sitostanol imply greater terrigenous input to the lake during and after the Initial Burning Period, as a consequence of increased erosion, reworking of burnt trees on the landscape as they slowly decompose, and runoff. At Lake Kirkpatrick, sitosterol and cholesterol fluxes were replaced by a rise in the reduced compounds sitostanol and cholestanol (Fig. 4) immediately after the Initial Burning Period, suggesting a high level of organic input resulting in reducing chemical conditions.

These erosion-related biomarkers were not present in Lake Diamond sediments, where all sterols and stanols increased between AD 1310 and 1380 (Fig. 2). The differences in water depth and catchment steepness between sites may result in lower sedimentation rate and thus limited reducing conditions at Lake Diamond. All sterol markers in Lake Kirkpatrick increased dramatically during the decades of European settlement (c. AD 1800-present), despite few or no changes in local fire activity and sedimentation rate. This increase is therefore ascribed mostly to the intensification of land use for sheep grazing^43^ and associated growing human presence in the Lake Kirkpatrick watershed. The lack of a 19^th^ century increase in sterols at Lake Diamond is consistent with the absence of sheep grazing and with the scarce anthropic pressure in this particular watershed.

## Human Colonization and Land-Use Activity in the South Island of New Zealand

Archeological and paleoenvironmental records indicate that Māori rapidly traveled throughout the North and South Islands of New Zealand soon after their arrival ~AD 1280^6–9,22^. North Island Māori populations cultivated a number of food crops, including kumara (*Ipomoea batatas*) and taro (*Colocasia esculenta*), which was likely associated with initial clearing with fire^49^. The inability to grow crops in the interior and southern South Island necessitated foraging for a wide array of forest, wetland and marine resources and the use of fire to promote key carbohydrate resources, such as fire-adapted bracken fern^50^.

Comparing the different proxies of humans, fire and vegetation reveals their relative source areas. The macroscopic charcoal and sterols data at Lake Kirkpatrick and Lake Diamond have a watershed source and jointly show the initial period of forest clearance (the Initial Burning Period) (Fig. 2) and the subsequent period of low-intensity or infrequent burning, and partial forest recovery c. AD 1500 to 1700 (Fig. 2a–e). The fire biomarkers – MAs and PAHs – show high levels of intense burning soon after human arrival, although their source area likely encompasses the whole South Island or beyond. The low charcoal influx and fire biomarker levels after the Initial Burning Period at L. Kirkpatrick suggest smaller and lower temperature fires until the arrival of Europeans. Not all peaks in fire intensity, recorded by PAH and levoglucosan (Fig. 2b,c), coincide with charcoal peaks (Fig. 1d) suggesting that the biomarkers may have a wider source area than the charcoal. Declines in fecal sterols flux (Fig. 2a) from c. AD 1400 to c. 1800 suggest reduced anthropogenic pressure in the watershed, even when forest clearance through fire was still occurring elsewhere on the island.

European settlement at the beginning of the 19^th^ century led to further forest conversion to scrubland and pasture in the South Island. However, fire biomarker concentrations do not significantly increase at the same time as pollen evidence of land use (Fig. 2b–d). The low levels of fire biomarkers, as compared to changes in pollen assemblages, imply that land-use practices did not require burning (i.e., grazing), the type of fuel shifted from forest to seral vegetation (including *Leptospermum*, bracken and grasses), or that the two proxies have different source areas.

The initial burning of forests by early Māori settlers therefore led to a dramatic transformation of the vegetation that continued again in the 19^th^ and 20^th^ centuries by Europeans. Recent size estimates of the initial founding group suggest it was composed of approximately 500 individuals^51^. It is remarkable that these small populations were able to convert c. 40% of New Zealand’s forests to grass and shrubland within 1–2 centuries of their arrival^7^. Our records of sterols and multiple fire markers, so closely matching paleoecological evidence, show that initial Māori presence in particular watersheds was brief and transient, and open vegetation was maintained by subsequent low-intensity fires and sporadic human use of the catchments until the arrival of Europeans. The successful demonstration of biomarker detection in New Zealand sediments, where the anthropogenic nature of fires is undisputed, illustrates the power of this approach to resolve the role of humans in biomass burning, in regions where fire drivers and the timing of human arrival are still debated.

## Methods

Two sediment cores were retrieved at Lake Kirkpatrick (195 cm) and Lake Diamond (160 cm) in 2009 using 7 cm polycarbonate tubes (Klein corer)^5,6^. The cores were split for archival purposes and the working half was sectioned into 1 cm thick slices. Three aliquots from each sample were processed separately for the analyses of charcoal, pollen (ref.^5,6^) and organic tracers (this study), respectively. Chronology was obtained by Accelerated Mass Spectrometry (AMS) ^14^C dates based on twig charcoal and plant macrofossils, and calibrated with BChron^5,6,52^.

72 samples were obtained in the 6–135 cm section of the Lake Kirkpatrick (0–191 cm) core and 49 samples were obtained for Lake Diamond (0–160 cm) in the 5–147 cm section. Wet samples were dried in the desiccator with silica gel until they maintained a constant weight and were then hand milled and homogenized in a ceramic mortar. Samples were stored at room temperature in sealed vials until extraction, which was performed with an ASE 200 (Accelerated Solvent Extraction, *Dionex Thermo Fisher Scientific*). Each sample was dispersed with diatomaceous earth and spiked with a known amount of a ^13^C-labeled internal standard solution for the quantification of the analytes (^13^C_6_-Cholesterol, ^13^C_6_-Acenaphtylene, ^13^C_6_-Phenanthrene, ^13^C_4_-Benzo(*a*)pyrene, ^13^C_6_-Levoglucosan) and extracted twice at 150 °C and 1500 psi with dichloromethane (L. Diamond) or with a dichloromethane:methanol = 9:1 (*v/v*) mixture (L. Kirkpatrick).

Extracts were concentrated in a centrifugal evaporator (Genevac EZ-2 Solvent Evaporator) up to ~0.5 mL and purified onto disposable solid phase extraction silica tubes (*Supelco* DSC-Si 12 mL, 2 g bed weight), previously conditioned with 40 mL of dichloromethane (DCM). The clean-up and fractionation of samples were achieved by eluting the samples with 70 mL of DCM followed by 20 mL of methanol (MeOH), adapting previously published procedures^24,46,53^. PAH and sterols were collected in the DCM fraction (F1), while MA were contained in the polar MeOH fraction (F2) and treated separately. F1 was concentrated to 100–200 μL and PAHs were analyzed through gas chromatography-mass spectrometry (GC-MS). After the analysis, 100 μL of BSTFA + 1% TMCS (N,O- Bis(trimethylsilyl)trifluoroacetamide + 1% Trimethylchlorosilane) were added to the samples to allow derivatization at 70 °C for 1 h. After a stabilization period of 24 h at room temperature, sterols were analyzed by GC-MS. F2 was evaporated to dryness, redissolved in 0.5 mL of ultrapure water and centrifuged before analysis by ion chromatography-mass spectrometry (IC-MS)^24^. The GC-MS and IC-MS methods, along with instrumental setup and target and qualifier mass-to-charge ratios employed are reported elsewhere^24,53,54^. Analytes were quantified based on internal standards through the isotope dilution technique. Results were calculated and corrected by the instrumental response factors, obtained by the repeated analysis of standard solutions containing all analytes and ^13^C internal standards at 100 pg µL^−1^ for PAH and 1 ng µL^−1^ for MA and sterols. Fluxes were calculated using the accumulation rate and the dry density following Menounos (1997)^55^.

Several procedural blanks were also analyzed in order to quantify possible contamination from the laboratory equipment. Absolute quantities were corrected for the blank plus three times the standard deviation and divided by the dry weight of samples to obtain concentrations. The average accuracy was 89 ± 16% for PAH, 115 ± 17% for sterols and 99 ± 11% for MA. Precision ranged between 2 and 23% for PAH, 12–24% for sterols 19–33% for MA.

The datasets generated during and/or analysed during the current study are available from the corresponding author on reasonable request.

## Electronic supplementary material

Supplementary information
