# Supplementary material for: Lake sediment fecal and biomass burning biomarkers provide direct evidence for prehistoric human-lit fires in New Zealand
Source: Sci Rep. 2018 Aug 14;8:12113. doi: 10.1038/s41598-018-30606-3 (PMC6092367; doi:10.1038/s41598-018-30606-3)
Supplement: Supplementary file 1 — Supplementary information [file 41598_2018_30606_MOESM1_ESM.pdf]

Title

Lake sediment fecal and biomass burning biomarkers provide direct evidence for prehistoric human-lit fires in New Zealand

Authors

E. Argiriadis[1*], D. Battistel[1,2], D.B. McWethy[3], M. Vecchiato[1], T. Kirchgeorg[1], N.M. Kehrwald[4], C. Whitlock[3], J.M. Wilmshurst[5,6], C. Barbante[1,2]

[1] Department of Environmental Sciences, Informatics and Statistics, Ca' Foscari University of Venice, Via Torino 155, 30170, Venezia Mestre (VE), Italy

[2] Institute for the Dynamic of Environmental Processes (IDPA-CNR), Via Torino 155, 30170, Venezia Mestre (VE), Italy

[3] Department of Earth Sciences and Montana Institute on Ecosystems, Montana State University, PO Box 173840, Bozeman, MT 59717, USA

[4] U.S. Geological Survey, Geosciences and Environmental Change Science Center, MS 980, Denver, CO 80225, USA

[5] Long-Term Ecology Lab, Landcare Research, PO Box 69040, Lincoln, New Zealand

[6] School of Environment, University of Auckland, Private Bag 92019, Auckland, New Zealand

* Corresponding author: elena.argi@unive.it

Supplementary information

Table S1 | List of considered polycyclic aromatic hydrocarbons with number of aromatic rings, weight class and mass-to-charge ratio used for the analysis.

**Compound**	**No. of aromatic rings**	**Weight class**[a]	***m/z***
Naphthalene	2	LMW	128
Acenaphthylene	2	LMW	152
Acenaphthene	2	LMW	154
Fluorene	2	LMW	166
Phenanthrene	3	MMW	178
Anthracene	3	MMW	178
Fluoranthene	3	MMW	202
Retene	3	MMW	234
Pyrene	4	MMW	202
Benzo(*a*)anthracene	4	MMW	228
Chrysene	4	MMW	234
Benzo(*b*)fluoranthene	4	HMW	252
Benzo(*k*)fluoranthene	4	HMW	252
Benzo(*a*)pyrene	5	HMW	252
Indeno(1,2,3-*cd*)pyrene	5	HMW	276
Benzo(*ghi*)perylene	6	HMW	276
Dibenzo(*ah*)antracene	5	HMW	278

[a] LMW: low molecular weight (128-166 g mol^{-1}), MMW: medium molecular weight (178-234 g mol^{-1}), HMW: high molecular weight (252-278 g mol^{-1}).

Table S2 | List of considered sterols with number of carbon atoms, origin and mass-to-charge ratio.

**Compound**	**No. of C atoms**	**Origin**	***m/z***
Coprostanol	C27	Human feces	215-370
Epi-Coprostanol	C27	Epimerization of Cop	215-370
Cholesterol	C27	Zoosterol	368-370
Cholestanol	C27	Zoosterol	355-460
Sitosterol	C29	Phytosterol	215-396
Sitostanol	C29	Phytosterol	215-473

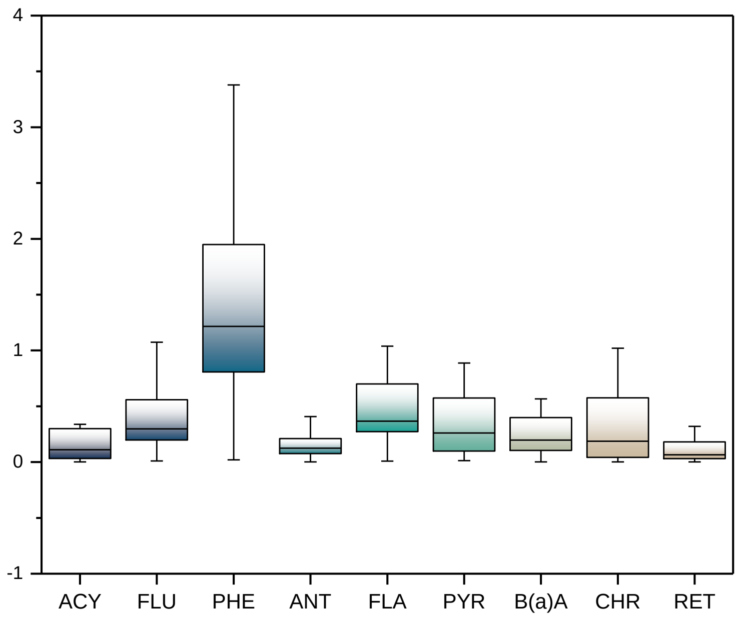

Figure S1 | PAH pattern. Distribution of PAH compounds detected in the sediment core from Lake Kirkpatrick.

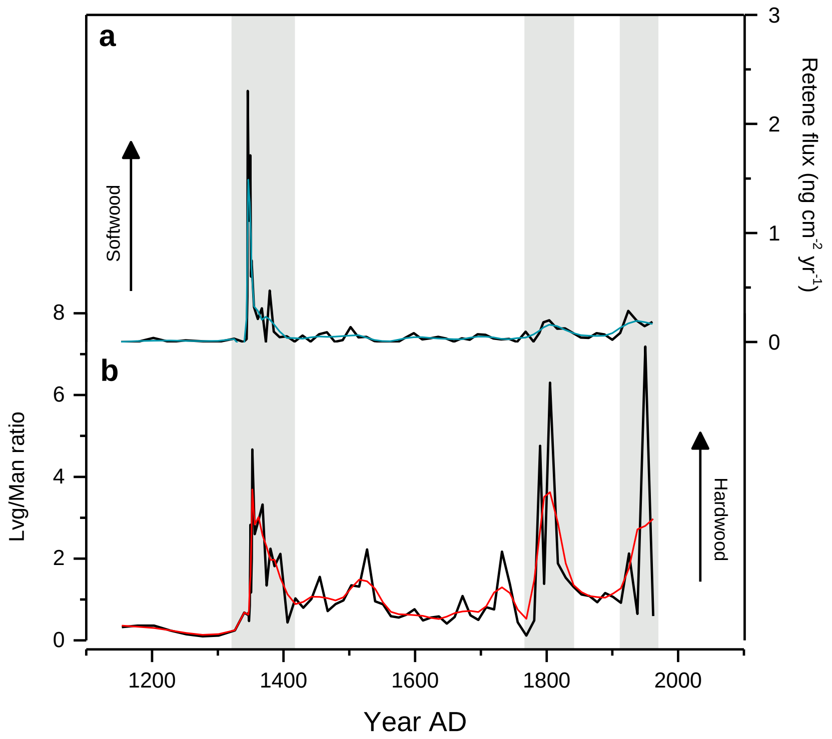

Figure S2 | Fuel characterization. a, Retene flux (ng cm^{-2} yr^{-1}) in Lake Kirkpatrick sediment core (black line) with 5 points adjacent averaging smoothing (blue line). **b**, Levoglucosan/mannosan ratio[1] in Lake Kirkpatrick sediment core (black line) with 5 points adjacent averaging smoothing (red line).

Sterols and stanols as terrigenous input and redox proxies

The use of Δ5-sterols and and 5α-stanols as markers of terrigenous input in sediments was first reviewed by Volkman (1986)[2]. The high content of sitosterol in plant tissue[3] makes this compound a good marker for the influx of organic material to the lake[4]. Moreover, the ratio between C29 and C27 sterols is frequently employed as indicator of terrestrial versus aquatic sources of lipids[2]. The degree of chemical conversion of Δ5-sterols (cholesterol and sitosterol) to 5α-stanols (cholestanol and sitostanol) was also suggested as a proxy of the redox conditions by several authors[5,6]. Conversion by anoxic bacteria is enhanced under highly reducing chemical conditions of the water column and the sediment, which may occur as a result of a high input of organic matter[6,7].

The high C29/C27 ratio in both the lake cores considered in this study (5÷10 for Lake Diamond and 2÷64 for Lake Kirkpatrick) indicates a clear predominance of allochthonous organic matter sources. The almost total absence of cholesterol in the Lake Kirkpatrick core and the abundance of cholestanol and sitostanol suggest the occurrence of anoxic processes[6] especially after the organic matter influx peak recorded at Lake Kirkpatrick during the Initial Burning Period.

References

1. Schmidl, C. *et al.* Chemical characterisation of fine particle emissions from wood stove combustion of common woods growing in mid-European Alpine regions. *Atmos. Environ.* **42,** 126–141 (2008).

2. Volkman, J. K. A review of sterol markers for marine and terrigenous organic matter. *Org. Geochem.* **9,** 83–99 (1986).

3. Meyers, P. a. & Ishiwatari, R. Lacustrine organic geochemistry—an overview of indicators of organic matter sources and diagenesis in lake sediments. *Org. Geochem.* **20,** 867–900 (1993).

4. Meyers, P. a. Applications of organic geochemistry to paleolimnological reconstructions: A summary of examples from the Laurentian Great Lakes. *Org. Geochem.* **34,** 261–289 (2003).

5. Vane, C. H. *et al.* Sedimentary records of sewage pollution using faecal markers in contrasting peri-urban shallow lakes. *Sci. Total Environ.* **409,** 345–56 (2010).

6. Nakakuni, M., Dairiki, C., Kaur, G. & Yamamoto, S. Stanol to sterol ratios in late Quaternary sediments from southern California: An indicator for continuous variability of the oxygen minimum zone. *Org. Geochem.* **111,** 126–135 (2017).

7. Ortiz, J. E. *et al.* Lipid biomarkers in Lake Enol (Asturias, Northern Spain): Coupled natural and human induced environmental history. *Org. Geochem.* **92,** 70–83 (2016).
